# Identifying and Prioritising BSACI Service Standards for Paediatric Allergy in the United Kingdom

**DOI:** 10.1111/cea.70176

**Published:** 2025-11-16

**Authors:** Paul J. Turner, Elizabeth Angier, Karen Brunas, Sarah Burrell, Max Chisholm, Emily Derrick, Matt Doyle, Catrina Drummond, Helen Evans‐Howells, Adam Fox, Mudiyur Gopi, Nasreen Khan, Susan Leech, Sian Ludman, Nick Makwana, Tom Marrs, Karina Montagni, Sophie Padua, Nandinee Patel, George Raptis, Ella Stoneham, Deepan Vyas, Amena Warner, Simon Williams, Katherine Cowan

**Affiliations:** ^1^ Imperial College Healthcare NHS Trust/Imperial College London London UK; ^2^ Portfolio GP and Research Student, Primary Care, Population Sciences and Medical Education University of Southampton Southampton UK; ^3^ Patient Representative, C/O BSACI London UK; ^4^ Lewisham and Greenwich NHS Trust London UK; ^5^ Island Medical Centre St Helier Jersey; ^6^ Dr Helen Allergy Ringwood UK; ^7^ Guy's and St Thomas' NHS Foundation Trust London UK; ^8^ National Allergy Strategy Group, C/O BSACI London UK; ^9^ Macclesfield District General Hospital/East Cheshire NHS Trust Macclesfield UK; ^10^ University Hospitals of Leicester NHS Trust Leicester UK; ^11^ Royal College of Physicians‐Improving Quality in Allergy Services (IQAS) London UK; ^12^ British Society for Allergy & Clinical Immunology London UK; ^13^ Royal Devon University Healthcare NHS Foundation Trust Exeter UK; ^14^ Sandwell and West Birmingham NHS Trust Birmingham UK; ^15^ Royal Hospital for Children Glasgow UK; ^16^ West Hertfordshire Teaching Hospitals NHS Trust Watford UK; ^17^ Allergy UK Crayford UK; ^18^ Anaphylaxis UK Surrey UK; ^19^ Katherine Cowan Consulting East Sussex UK

**Keywords:** allergic disease, children, James Lind Alliance prioritisation, service standards

## Abstract

**Background:**

Demand for paediatric allergy services has risen significantly over the past 20 years. National health datasets suggest almost 40% of children have an allergy diagnosis. Existing service standards from the Royal College of Paediatrics and Child Health (RCPCH) have focused on specific disease care pathways and the interface between primary and secondary care services. Given strategic changes to NHS children and young people's services, we undertook a collaborative project between RCPCH and the British Society of Allergy and Clinical Immunology (BSACI) to define service priorities for Paediatric Allergy Care accreditation in the UK.

**Methods:**

A BSACI working group developed 29 potential service standards. A consultation was then undertaken amongst BSACI members to identify indicative priorities. Potential standards were then prioritised using methodology adapted from the James Lind Alliance, by 24 stakeholders representing patients/patient groups (9), commissioners (2) and healthcare professionals (13).

**Results:**

Seventeen strategic priorities were identified, under the following 6 themes: service delivery, prevention, diagnostics, management, communication, maintaining standards/education. These included: setting a minimum staffing mix for multidisciplinary teams providing paediatric allergy care (medical specialist(s), specialist nurse, dietitian, health psychologist); prompt recognition and management of complex, multisystem allergic disease; working within a regional network to ensure access to specialist paediatric allergy services; use of integrated care pathways and supporting primary care accordingly; supporting early access to interventions proven to reduce the risk of atopic disease (with consideration to potential barriers to access, including language, cultural, socioeconomic factors and other communication barriers); offering a referral pathway for disease‐modifying treatment where appropriate and effective transitioning to adult care.

**Conclusions:**

These priorities form a basis for the delivery of high‐quality care to children and young people affected by allergic disease.

## Graphical abstract

A total of 29 potential service standards for paediatric allergy were developed. These were then prioritised using methodology adapted from the James Lind Alliance, by stakeholders representing patients, service commissioners and healthcare professionals. The final 17 strategic priorities form a basis for the delivery of high‐quality care to children and young people affected by allergic disease.

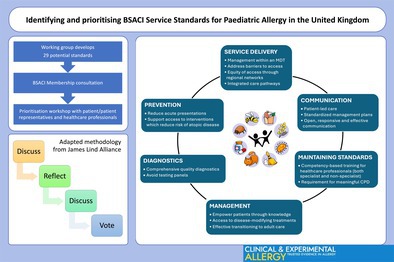

**Abbreviations**
BSACI: British Society of Allergy and Clinical Immunology
CPD: continuing professional development
CYP: children and young people
JLA: James Lind Alliance
MDT: multidisciplinary team
PREMs: patient‐related experience measures
PSE: priority setting exercise
PSP: priority setting partnership
QoL: quality of life
RCPCH: Royal College of Paediatrics and Child Health

## 1 Introduction

Allergic diseases (including eczema, asthma, allergic rhinitis and food allergy) are amongst the most common chronic conditions in Western society, with a lifetime prevalence of up to 30% in the UK [1]. Prevalence increased at the end of the 20th century, and national primary care datasets suggest that almost 40% of children now have an allergy diagnosis [2, 3].

In 2006, the UK Department of Health commissioned the Royal College of Paediatrics and Child Health (RCPCH) to develop care pathways for children and young people (CYP) with allergies [4]. Eight disease‐specific care pathways were published in 2011, with a focus on the interface between primary and secondary care services [4]. At the same time, it was acknowledged that changes in service commissioning alongside increased demand risked specialty networks and pathways of care becoming fragmented and disjointed. As a result, work was undertaken to provide clearer descriptions of specialist services. This resulted in the publication of national service standards for paediatric allergy in 2013 [5], using a national template of five overarching domains of care that had been developed to apply to all specialities (see Table 1).

**TABLE 1:** Summary of 2013 NHS service specifications for specialised paediatric allergy services.

Domain	General outcome	Specific outcome
Preventing people from dying prematurely	Minimise mortality by providing the most appropriate care for children with allergic disease.	
Enhancing quality of life for people with long‐term conditions	Minimise morbidity by providing the most appropriate care for children with allergic disease. Work with secondary care network providers to ensure high quality allergy services at secondary care level.	Reduction in time away from school due to ill health, by improved management of allergic conditions Improvement in QoL for children receiving immunotherapy for pollen or food allergy Fewer allergic reactions and unscheduled healthcare service visits through appropriate management of children with complex allergic disease Reduction in unscheduled healthcare visits and improvement in QoL through appropriate management of children with severe eczema
Helping people to recover from episodes of ill‐health or following injury		Documented improvement in symptoms and reduction in medication use for children receiving immunotherapy with inhalant allergens.
Ensuring people have a positive experience of care		Documenting and acting on deficiencies identified through the use of the RCPCH allergy‐specific PREMS on an annual basis. Minimise the potential disruption of care and stress for older children and their parents/carers through the provision of organised transition to an adult allergy service
Treating and caring for people in safe environment and protecting them from avoidable harm	Ensure there is sufficient, skilled and competent multi‐disciplinary workforce to manage children with allergic disease. Ensure that children with allergic disease are treated in line with national guidelines and agreed local pathways.	Minimise the risks to children by providing immunotherapy, immunomodulatory treatments and high‐risk challenges in a safe environment, in accordance with national and international best practice

Abbreviations: PREMs, patient‐related experience measures; QoL, quality of life.

Some challenges were experienced in applying these domains to long‐term conditions such as allergic disease. For example, deaths from allergic diseases such as food allergy are fortunately rare; thus, applying the general aim of “preventing people from dying prematurely” as an outcome measure or metric is difficult to implement, since so few people experience fatal outcomes from food allergy [6, 7]. Arguably, reducing the morbidity and adverse impact on quality of life is a more pragmatic outcome. A further challenge is the liminal nature of allergic disease. ‘Liminality’ represents a “state” between illness and wellness: despite having an allergic diagnosis, people with allergies do not consider themselves fully ‘ill’ or entirely ‘well’, but something in between [8]. For example, individuals with IgE‐mediated food allergy are typically “well” so long as they avoid their trigger food allergen(s)—but failure of dietary avoidance can rapidly cause acute, even critical, deterioration [9]. Liminality is important in CYP, because their diagnosis sets them apart, impacting their self‐perception and social interactions which can lead to denial and adverse health outcomes. This can profoundly influence self‐management, treatment adherence, and emotional wellbeing [8]. The impact of liminality was not fully appreciated when the current service standards were developed in 2013.

The RCPCH 2040 plan emphasised the importance of integrated care, giving an opportunity to review the delivery of allergy services and develop new models of patient‐centred care—the aim being to deliver outcomes‐based commissioning with quality measures embedded in delivery [10]. With this in mind, we undertook a collaborative project between RCPCH and the British Society of Allergy and Clinical Immunology (BSACI) to develop accreditation standards for the delivery of Paediatric Allergy Care in the NHS. As part of this project, we set out to define priorities in the delivery of allergy care in the UK. Clinical and research priorities are often developed without wide and coordinated stakeholder input [11]. To address this, the James Lind Alliance (JLA) has developed a methodology for Priority Setting Partnerships (PSPs), which brings together patients, carers and clinicians to identify and prioritise the evidence uncertainties in any given topic area [12]. We therefore undertook a Priority Setting Exercise (PSE), adapting PSP principles and drawing on elements of the Delphi method of consensus development, to focus on service delivery and identify key priorities in developing national service accreditation standards for paediatric allergy care in the UK.

## 2 Methods

The PSE was undertaken in 4 stages:

- *Initiation and identification of potential stakeholders*: a project group was established to oversee the PSE activity and identify potential stakeholders. The project group included healthcare professionals involved in the multidisciplinary care of CYP with allergies (with representation from primary and secondary/tertiary care, and devolved nations) and patient charity groups (Allergy UK, Anaphylaxis UK). While a large proportion of CYP with allergies have concomitant asthma, we did not recruit stakeholders representing asthma alone because there are already established service standards and standards of care for asthma.
- *Identifying potential strategic standards*: the project group used the 2011 RCPCH pathways [4] and 2013 NHS Service Standards [5] to identify potential standards within the following 6 themes: prevention, diagnostics, management, communication, access to services, education (Table 2).
- *Feedback and indicative prioritisation*: a national survey of BSACI members was undertaken in November 2023 to refine the potential standards and suggest any additional items. Using a 5‐point Likert scale, respondents rated each item as to how important it was as a key standard for service accreditation.
- *Prioritisation workshop*, where the standards identified in (ii) were prioritised through consensus amongst 24 stakeholders, representing patients/patient groups and healthcare professionals (Table S1). HCPs were recruited through the BSACI Membership survey, while patients/caregivers were recruited through the Project Group. All participants were asked to declare any interests prior to the workshop. Explanatory notes (see online appendix) were used to brief participants prior to the workshop. Workshop participants separately undertook the same rating exercise used in the national BSACI membership survey (iii) to rate the importance of each standard as a key standard for service delivery (and thus to inform accreditation) using a 5‐point Likert scale. This was done anonymously and prior to sharing the results from the membership survey. The results from both surveys were then used to inform the prioritisation exercise (based on PSP principles and drawing on elements of the Delphi method of consensus development) to focus on service delivery and identify key priorities in developing national service accreditation standards for paediatric allergy care in the UK. The workshop was held online in March 2024. Items required at least 70% consensus to be included for prioritisation.

**TABLE 2:** Items considered for prioritisation.

*Service impact and evidence base*	1^a^	2^b^
Use of validated tools (such as instruments to assess QoL) to monitor impact of interventions and generate evidence base to inform future policy decisions	3.58	3.36
Transparent governance processes in place including audit of service delivery	3.91	4.14
*Prevention*		
Support early access to interventions proven to reduce risk of atopic disease	4.25	4.14
Management strategies to reduce acute episodes/presentations	4.19	4.42
*Diagnostics*		
Access to high quality and comprehensive diagnostics	4.40	4.29
Reducing risk of unvalidated diagnostic approaches	4.14	3.79
Prompt recognition of multisystem allergic disease	4.26	4.07
Recording of anthropometric data (height/weight and in under 2s, head circumference) to monitor nutritional status and control of chronic disease	3.89	3.5
*Management*		
High quality training for self‐management (including medical devices)	4.63	4.21
Management of patients within a defined minimum multidisciplinary team	4.56	4.36
Embedding safeguarding principles into specialist allergy provision	3.82	3.93
Offering referral pathway for disease‐modifying treatment where appropriate	4.25	4.14
Effective transitioning pathway to adult care	4.12	4.43
Supporting workforce development, functioning and wellbeing	4.02	3.64
*Communication*		
Family/patient‐led care with shared decision‐making	4.39	4.21
Open, responsive and effective communication with other stakeholders	4.02	3.79
Use of standardised allergy action plans	4.35	4.21
Correct documentation of allergies (not just drug) on all relevant systems	4.19	4.29
Ability for staff and service users to provide feedback confidentially	3.88	3.71
*Access to services*		
Development of integrated care pathways to facilitate access to allergy services and achieve appropriate prioritisation of referrals	4.18	4.57
Poverty proofing (considering vulnerable groups & social determinants of care)	3.88	4.07
Access to other specialist services for complex multisystem allergic disease	4.37	4.07
Working within a regional network to ensure access to specialist services	n/a	4.21
*Education*		
Standardised minimum training for specialist allergy (medical/nursing/dietetic) staff	4.37	4.57
Minimum requirement for multidisciplinary team staff to maintain specialist status through allergy‐related continuing professional development (CPD)	4.26	4.43
Empowering patients/public through knowledge/training	4.07	4.00
Providing training for non‐specialist HCPs to support integrated care model	3.88	4.00
Training of students/trainees in recognition and management of allergies	n/a	4.36
Links with relevant clinical research teams	n/a	3.86

^a^ Score 1 = Weighted average (out of 5) from survey of BSACI Members, November 2023.

^b^ Score 2 = Weighted average (out of 5) from survey of workshop participants, February 2024.

## 3 Results

Twenty‐six potential service standards were developed by the Project group (see Table 2), and this formed the basis for the BSACI membership consultation. Responses were received from 61 members, representing specialist paediatric allergists (30%), specialist allergy nurses (15%), general paediatricians (38%), dietitians (5%), specialist adult allergists (5%) and other (7%). The indicative scores from the consultation are shown in Table 2. Comments from the consultation were considered by the Project group, resulting in the addition of a further 3 items: working within a regional network to ensure access to specialist services; training of students/trainees in recognition and management of allergies; links with relevant clinical research teams. The pre‐workshop survey by participants included these additional items (Table 2).

The final items prioritized for inclusion in service standards for Paediatric Allergy are listed in Table 3, and could be summarized into 6 themes: service delivery, prevention, diagnostics, management, communication, maintaining standards/education. The following did not achieve the 70% threshold to be included: Transparent governance processes including audit of service delivery (62%); Reducing risk of unvalidated diagnostic approaches (38%); Supporting workforce development, functioning and wellbeing (12%); Correct documentation of allergies (not just drug) on all relevant systems (54%); Poverty proofing (4%); Training of students/trainees in recognition and management of allergies (38%); Research links (21%). Notably, participants did not conclude these items are unimportant, but rather, should not be prioritized above items which were chosen.

**TABLE 3:** Priority items in paediatric allergy service delivery.

	% consensus
*Service delivery*	
Management of patients within a multidisciplinary team (MDT) consisting, at a minimum, of:	100%
– Medical specialist(s) (with specialist training in Paediatric Allergy) – Specialist nurse(s) – Dietitian(s) – Access to health psychology – Access to other specialist services as needed, to ensure prompt recognition and management of complex, multisystem allergic disease (e.g., Dermatology, Gastroenterology, Respiratory)	88%
Consider the impact of potential barriers to access (e.g., language, cultural, socioeconomic factors) for both management and prevention.	100%
Working within a regional network to ensure access to specialist paediatric allergy services throughout the UK	88%
Develop integrated care pathways to facilitate access to allergy services *and achieve appropriate prioritisation of referrals*	100%
*Prevention*	
Management strategies to reduce acute episodes/presentations (and reduce time out of education/work)	100%
Support early access to interventions proven to reduce risk of atopic disease (*with consideration to potential barriers to access*)	92%
*Diagnostics*	
Access to high quality and comprehensive diagnostics (underpinned by appropriate benchmarking/quality control/Standard Operating Practices)	100%
*Management*	
High quality training for patients/caregivers to self‐manage allergic disease (including medical devices) to empower patients through knowledge	100%
Offering referral pathway for disease‐modifying treatment where appropriate	88%
Ensuring effective transitioning pathways to adult care	100%
*Communication*	
Patient/family‐led care with shared decision‐making	100%
Use of standardised allergy action plans such as those produced by BSACI	71%
Open, responsive and effective communication with other stakeholders (including with Primary Care)	71%
*Maintaining standards/education*	
Defined and standardised minimum, competency‐based training for healthcare professionals working in specialist paediatric allergy	100%
Minimum requirement for staff to maintain specialist status through allergy‐related continuing professional development (CPD)	100%
Provide training to support non‐specialist healthcare professionals in delivery of integrated care	79%
Empowering patients/public through knowledge/training	74%

## 4 Discussion

There has been recognition at the highest levels of government as to the deficiencies in allergy care in the UK [13, 14]. However, this recognition has not translated to improvements in service delivery [15]. Indeed, the DoH refused to endorse the 2011 RCPCH care pathways for CYP with allergies, despite funding their development [16]. Around half of atopic children have multi‐organ allergic disease [3]; these patients may best be managed by a single specialist team able to provide more holistic care and better address the psychosocial aspects of disease management. But there are increasing disparities in allergy care provision, with a mismatch between the location of specialist (“tertiary”) allergy services in the UK and the population who require them [17, 18, 19]. The majority of outpatient appointments for allergic disease in CYP are with a general paediatrician with a specialist allergy interest, and not an allergy specialist [17]. Whilst this may be appropriate for many patients, RCPCH data suggest that the majority of consultants providing allergy care have not received specialist training in allergy, for example through the RCPCH Special Interest (SPIN) modules. This is just the tip of the iceberg: the vast majority of children with allergic diseases are only seen within primary care. For example, over 97% of NHS consultations for food allergy occur in primary care, even in those with previous anaphylaxis [18]. This justifies the importance of integrated care pathways with multidisciplinary approaches to improve allergy care [16].

A critical element flagged by participants was the importance of ensuring equity of access to high‐quality allergy care from appropriately trained healthcare professionals throughout the UK. As one respondent put it, “we are past the days where we see allergy as a single system disease; patients should not be subjected to a postcode lottery for standardised quality care.”

With respect to those items not included in the final prioritisation, the following comments were made: Participants acknowledged that while the priority must be to deliver excellent clinical care to patients, they did not feel that a requirement to use validated tools and instruments to monitor impact on Quality of Life as a matter of clinical routine was justified. Some items (such as those relating to governance, service feedback or documentation of patient anthropometric data) were considered mandatory within broader NHS Service delivery, and therefore not required for inclusion in paediatric allergy‐specific service specifications.

Although “poverty‐proofing” services was important, participants did not rate this item as a specific priority. Reasons for this included the need to poverty‐proof paediatric services more broadly, as outlined in the RCPCH 2040 plan [10]. Some participants flagged that paediatric allergy may have specific issues, with a perception that some socioeconomic groups are better able to vocalise the perceived needs of their children than others [19]. A further issue is the role of lifestyle interventions (rather than the use of medications alone) in managing allergic disease, which makes some interventions more challenging for lower socioeconomic groups. As a result, there was broad agreement that service delivery should consider the impact of potential barriers to access (e.g., language, cultural, socioeconomic factors), particularly with respect to strategies proven to reduce the risk and/or severity of allergic disease. There was also recognition that vulnerable groups include those with learning difficulties/disabilities (both CYP and their parents) who must have their needs taken into account.

Service standards require monitoring and audit to ensure implementation. Participants were concerned that the addition of mandatory audits beyond those already required by NICE Quality Standards might increase the administrative burden on services without leading to an improvement in clinical outcomes. There was broad support for benchmarking through peer‐monitoring: where clinical staff from other services would visit other clinics to both monitor accreditation standards and facilitate shared learning with respect to service delivery [20]. Such a strategy would also be aligned with methods to deliver meaningful quality improvement [21]. At the same time, there must be acknowledgment that “no one is better qualified to comment upon the quality of care provided by a paediatric centre than the children and young people receiving that care” [22]. It is therefore critical that patient experience is included in any service evaluation and accreditation process. To this end, RCPCH has already developed an allergy‐specific patient‐reported experience measure (PREM) that can be used for this purpose [23].

Paediatric Allergy is a relatively “young” subspecialty which is undergoing a paradigm shift in service delivery, from a predominantly diagnostic service to a therapeutic service where disease‐modifying treatments are becoming the standard of care. The benefits of having service standards are listed in Table 4. Given current variability in how paediatric allergy care is delivered across regions, ensuring consistent and high‐quality clinical care as we move into a new phase of therapeutics is critical. Education is clearly going to be a fundamental requirement: the 2011 RCPCH care pathways included a listing of the required competences for healthcare professionals at all levels [4]. We agree with previous conclusions: that “harmonization of allergy components within undergraduate curricula is crucial to ensure all physicians develop the appropriate allergy‐related knowledge and skills, particularly in light of inconsistencies seen in the primary care management of allergy” [24]—something which should be addressed in the upcoming UK National Allergy Strategy [25]. Our hope is that the priorities identified here will inform the future delivery of care to children and young people affected by allergic disease, as the BSACI works with other stakeholders to provide a basis for service accreditation in the NHS.

**TABLE 4:** Benefits of service standards.

Consistency	It ensures that all accredited clinical services meet a set standard of quality and care, promoting consistency in service delivery across different regions and healthcare providers.
Quality improvement	By establishing clear standards, the framework provides a benchmark for clinical services to measure themselves against, encouraging ongoing quality improvement efforts.
Patient safety	Standardised practices can enhance patient safety by reducing the likelihood of errors or substandard care. This is particularly important in paediatric allergy where accurate diagnosis and appropriate management are crucial.
Transparency	Having a transparent framework allows patients and their families to understand what to expect from accredited clinical services, empowering them to make informed decisions about their care.
Efficiency	Standardised processes and procedures can lead to greater efficiency in service delivery, improving patient experience and potentially reducing wait times and improving access to care.
Professional development	The framework may include requirements for ongoing education and training for healthcare professionals involved in paediatric allergy care, supporting their continuous professional development.
Research and Innovation	A standardised framework can facilitate data collection and research efforts, leading to advancements in the understanding and treatment of paediatric allergies.

## Author Contributions

Paul J. Turner drafted the initial draft. The draft was then circulated for comment amongst all co‐authors. All co‐authors approved the final draft and the decision to submit for publication.

## Conflicts of Interest

Paul J. Turner reports grants from the UK Medical Research Council, NIHR/Imperial Biomedical Research Centre and UK Food Standards Agency, outside the submitted work; he is vice‐chair of the National Allergy Strategy Group (NASG), and a member of the BSACI Paediatric Allergy Committee (PAC). Elizabeth Angier has received research funding from the Natasha Allergy Research Foundation outside the submitted work and is a member of the BSACI Primary Care Committee and the NIHR School for Primary Care Research. Matt Doyle has received speaker expenses from DBV Technologies in 2024, is previous Chair of the BSACI Primary Care Committee and a current member of the BSACI Standards of Care committee. Adam Fox is Chair of NASG and previous Chair of the Allergy UK Health Advisory Board; Independent Chair of Data Monitoring Committee for commercial research sponsored by ALK‐Abello. Mudiyur Gopi is a member of the BSACI PAC and NASG, representing secondary care services. Helen Evans‐Howells is a trustee for Anaphylaxis UK, a patient charity; has received payment from Mylan/Viatris, Thermofisher, Astra Zeneca, Abbott, Danone, Nutricia, Nestle Health Science and Mead Johnson to provide GP education; consulting fees from Grow With Iris, Viatris, Nestle Health Science, ALK; university fee support from Nutricia and Mead Johnson; funding from the Sadie Bristow Foundation for delivering allergy care. Nasreen Khan is clinical lead for Improving Quality in Allergy Services (IQAS) accreditation programme through the Royal College of Physicians. Susan Leech is co‐chair of the BSACI Standards of Care committee. Nick Makwana is chairperson of the BSACI PAC. Tom Marrs is current chairperson of the BSACI PAC, and has received speaker expenses from DBV Technologies and provided consulting to Thermofisher, outside the submitted work. Nandinee Patel is a member of the BSACI PAC and the NHS‐England Specialist Allergy Group. Katherine Cowan was senior adviser to the James Lind Alliance and coauthor/editor of the JLA Guidebook from 2008 to 2023. She was an independently contracted adviser for this work. BSACI has had partnerships with the following companies or institutions from January 1st to December 31st, 2024: Alk Abello, AstraZeneca, Dr. Falk Pharma, Glenmark, Viatris, Allergy team, Allergy UK, ARS Pharma, Bio‐Diagnostics Ltd., Captium, DBV technologies, Dermal, Diagenics, EOS Network, IQAS, KalVista, L'Oreal, Mast Cell Action, NeilMed, Novartis, Pharming, Scope Ophthalmic, Stallergenes Greer, Aimmune, Initiative, Group CCM. The other authors declare no conflicts of interest.

## Supporting information


**Table S1:** Stakeholders at the prioritisation workshop.

## Data Availability

All available data (not restricted due to data protection regulation) is included in the main manuscript and Supporting Information.
