# Supplementary material for: Identifying and Prioritising BSACI Service Standards for Paediatric Allergy in the United Kingdom
Source: Clin Exp Allergy. 2025 Nov 16;56(1):7–14. doi: 10.1111/cea.70176 (PMC12774573; doi:10.1111/cea.70176)
Supplement: Supplementary file 1 — Table S1: Stakeholders at the prioritisation workshop. [file CEA-56-7-s001.doc]

SUPPORTING INFORMATION

**Table S1**: Stakeholders at the Prioritisation Workshop

| Elizabeth Angier | GP and Primary care lead for NHS Hampshire & Isle of Wight Integrated Commissioning Board (ICB). |
| --- | --- |
| Karen Brunas | Parent of a young person with lived experience of allergic disease and anaphylaxis. Member of Independent Data Monitoring Committee for Food allergy Immunotherapy studies. Local representative for Anaphylaxis UK. |
| Max Chisholm | Young person with lived experience of allergic disease and anaphylaxis. |
| Judith Clayton | GP and member of BSACI Standards of Care committee. |
| Emily Derrick | Consultant in Paediatric Allergy and General Paediatrics, Homerton Hospital NHS Trust. |
| Matt Doyle | GP; Chairperson, BSACI Primary Care Committee. |
| Catrina Drummond | Parent of a young person with lived experience of allergic disease and anaphylaxis. |
| Helen  Evans-Howells | GP; Chair of the Anaphylaxis UK Clinical and Scientific Panel |
| Adam Fox | Consultant in Paediatric Allergy, Guys and St Thomas’ NHS Foundation Trust; Chair, National Allergy Strategy Group. |
| Mudiyur Gopi | General Paediatrician with Specialist interest in Allergy, Macclesfield. |
| Nasreen Khan | Consultant in Adult Allergy; IQAS clinical lead, Royal College of Physicians. Member of BSACI Standards of Care committee. |
| Susan Leech | Consultant in Paediatric Allergy, Kings College Hospital; co-chair, BSACI Standards of Care committee |
| Sian Ludman | Consultant in Paediatric Allergy, Exeter. |
| Nick Makwana | Consultant in Paediatric Allergy; Chairperson, BSACI Paediatric Allergy Committee |
| Kate Mayer | Commissioning team, Nottinghamshire and Nottingham ICB. |
| Karina Montagni | Parent of a young person with lived experience of allergic disease and anaphylaxis. |
| Sophie Padua | Young adult with lived experience of allergic disease and anaphylaxis. |
| Nandinee Patel | Consultant in Paediatric Allergy; Member, NHSE Specialist Allergy Service |
| Simone Miles | CEO, Allergy UK. |
| George Raptis | Consultant in Paediatric Allergy, Glasgow |
| Ella Stoneham | Young person with lived experience of allergic disease and anaphylaxis. |
| Deepan Vyas | General Paediatrician with Special interest in Allergy, Watford |
| Amena Warner | Head of Clinical Services, Allergy UK. |
| Simon Williams | CEO, Anaphylaxis UK. |

Explanatory Notes provided to workshop participants

| **SERVICE IMPACT and EVIDENCE BASE** |
| --- |

| Use of validated tools (e.g health-related quality of life [HRQL] instruments, asthma control questionnaire/test [ACQ/ACT]) to monitor impact of interventions and generate evidence base to inform future policy decisions |
| --- |

- Questionnaires have been developed to assess the impact of asthma/eczema/hay fever/food allergy on patients or their parents.
- Is it important to use these to formally monitor treatments, and see if they work for patients? If so, is this “best practice” or a key standard?

| Transparent governance processes in place including audit of service delivery |
| --- |

- There is a document which outlines the scope of the service provided (including who the service aims to provide treatment/care for, and whether research or training is undertaken), the range of services offered, information about service delivery and how referrals are managed.
- The clinical service has a procedure in place outlining how incidents, adverse events and ‘near-misses’ are reported and investigated.
- An audit of the service is undertaken on an annual basis against key performance indicators (KPIs). This is used to plan for service improvement and development (responsive to local needs) at governance meetings, which are formally minuted.

| **PREVENTION** |
| --- |

| Support early access to interventions proven to reduce risk of atopic disease (with consideration to potential barriers to access e.g. language, cultural etc.) |
| --- |

- Patient care must be proactive: prevention of allergic diseases is key and should include measures to reduce the risk allergy (e.g. timing of introduction of solid foods into the infant diet in children at risk of food allergy) as well as appropriate provision of rescue medication in line with national guidelines.
- Strategies must be implemented to overcome local barriers to preventative measures, and be sensitive to the individual needs of families. This will involve closer working with Primary Care and Health Visitors to increase knowledge and understanding of prevention strategies.

| Management strategies to reduce acute episodes/presentations (and reduce time out of education/work) |
| --- |

- Preventing ill health should be of primary importance, therefore patient care should be targeted towards minimising the impact of allergic disease on health and daily life.
- Strategies should also include ways to minimise time-off school/ education/work – for example, reducing hospital visits (e.g. through remote consultations) can reduce time off-work for parents

| **DIAGNOSTICS** |
| --- |

| Access to high quality and comprehensive diagnostics |
| --- |

- Incorrect diagnosis can occur with food allergy, because tests detect the antibody which causes food allergy, which does not always match whether a child/young person actually has the food allergy.
- It is therefore important that patients are offered high quality and comprehensive diagnostics, to avoid the situation where patients are put on to unnecessary dietary restrictions (e.g. due to use of testing panels).
- Includes access to accredited laboratories, to ensure quality control.

| Reducing risk of unvalidated diagnostic approaches |
| --- |

- Access to quality diagnostics and their correct interpretation has resulted in the availability of unvalidated diagnostic approaches outside the NHS, often marketed by individuals with no appropriate training or knowledge.
- Is there a role for clinics to reduce the risks associated with unvalidated diagnostic approaches?

| Prompt recognition of multisystem allergic disease (in collaboration with other specialist services), leading to referral where needed. |
| --- |

- Children/young people affected by allergies often have multiple allergic diseases, which can require input from other healthcare professionals, for example, working in respiratory medicine (asthma), gastroenterology (allergic gut disease), ophthalmology (allergic eye disease).
- Allergy clinics should therefore have links with other specialist services, and ensure early recognition when their involvement is needed for optimal patient care.

| Recording of anthropometric data (height / weight / head circumference in <2yrs) to monitor nutritional status and control of chronic disease |
| --- |

- Growth is a key measure of health in children/young people, particularly those with severe allergic disease ± multiple food allergies.

| **MANAGEMENT** |
| --- |

| High quality training for self-management (including devices) |
| --- |

- Self-care and self-management is key to managing allergic disease, and it is important that children/ young people are involved in management, including use of medicines and monitoring the allergies.
- Devices (including medical devices such as inhalers, auto-injectors) and monitoring devices (such as peak flow meters) are commonly used in those with allergies, but require specific and age-appropriate training.

| Management of patients within MDT (with a defined minimum team) |
| --- |

- Working within a multidisciplinary team (MDT) is critical and is very likely to improve patient outcomes, although not every allergic child/ young person needs to be managed by an MDT.
- The MDT should include access to dietetic support and, where needed, a health psychologist to reduce impact of severe disease.

| Embedding safeguarding principles into specialist allergy provision |
| --- |

- Safeguarding is critical in all aspects of child health, including allergy services.
- Should services have specific safeguarding provisions with regular safeguarding supervision sessions, in order to be accredited? Or is this not needed, as it should already be provided through general clinical care provision?

| Offering referral pathway for disease-modifying treatment where appropriate (including access to new therapies) |
| --- |

- New disease-modifying treatments such as “biologics” and “immunotherapy” are being introduced into the NHS. Accredited clinics must offer these treatments to their patients in line with national guidelines, or have clear referral pathways to more specialist clinics in their Network where these treatments can be given.
- Referral pathways must include consideration as to equity of access on the basis of clinical need.

| Effective transitioning pathway to adult care |
| --- |

- Transition is the process of preparing, planning and moving young people from children’s services to their GP or local adult services.
- There should be clear criteria and referral pathways to local adult services (both allergy and other specialties) in line with national guidelines for transitioning of patients with allergies.

| Supporting workforce development, functioning and wellbeing |
| --- |

- Systems should be in-place to monitor MDT functioning and wellbeing, for example through a staff satisfaction survey and 360-feedback. Where the MDT includes a health psychologist, they should have a key role in supporting workforce development as part of their job description.
- There should be allowances to ensure (allergy-specific) training to meet the “continuing professional development” (CPD) requirements for individual MDT members.

| **COMMUNICATION** |
| --- |

| Family/patient-led care with shared decision-making |
| --- |

- Shared decision-making ensures that individuals are supported to make decisions that are right for them. It is a collaborative process through which a clinician supports a patient to reach a decision about their treatment.
- Shared decision-making encourages patients and families to take a more central and active role in their care by working with their doctor to participate in the choices surrounding their treatments and medication options.
- Children/young people must be involved in shared-decision making. This is particularly important with allergies, where self-care is a critical part of management. Their views should be documented on clinical notes.
- Services should consider use of an “allergy passport”, particularly with transitioning of young adults out of children’s services.
- Does this include “Empowering patients/public through knowledge/ training” or should this be separate

| Open, responsive and effective communication with other stakeholders *(including GPs and Primary Care staff)* |
| --- |

- Access to responsive care (there is a systemin place so patients/ families can obtain support in between appointments from MDT members) rather than having to wait for annual reviews etc.
- Communication must be effective, both within the MDT and to others outside the MDT including patients and their families (and thus take into account language accessibility).

| Use of standardized allergy action plans |
| --- |

- Services must use national standardized action plans, e.g. BSACI Action Plan for food allergy.

| Correct documentation of allergies (not just to drugs) on all relevant systems |
| --- |

- While declaration of drug allergies is an NHS standard, this does not apply to non-drug allergies.
- Service accreditation must include complete documentation of all allergies affecting patients in their care.

| Ability for staff and service users (including CYP) to provide feedback confidentially |
| --- |

- There must be a documented and transparent process to allow service users and staff to provide feedback in confidence.
- Service users should be encouraged to make comments on improvements to the service in ways that are readily available and accessible (for example, posters in clinic with clear signage or leaflets readily accessible to patients).
- Any action taken or improvements made in response to service users’ views should kept on record and shared with users who provided feedback or raised concerns.

| **ACCESS to SERVICES** |
| --- |

| Development of integrated care pathways to facilitate access to allergy services *and achieve appropriate prioritisation of referrals* |
| --- |

- There must be clear pathways and referral criteria to help triage and streamline referrals from primary care (GPs). Patients should only be referred when indicated by an allergy-focused clinical history and (where appropriate) diagnostic testing (to avoid inappropriate referrals)
- Rapid referral pathways for infants to be seen as needed (food allergy, eczema, wheezing)
- Services should consider how to provide advice/guidance/support to community services, ensuring equity of access to specialist allergy services.

| Poverty proofing (with consideration to vulnerable groups/social determinants) |
| --- |

- Minimising the impact of poverty on healthcare provision is key to breaking the link between an individual’s income and their opportunity to live a long, healthy life.
- According to the King’s Fund, there are 3 key roles for the NHS: raising awareness among staff, developing concerted action to meet the needs of those experiencing poverty and using its voice to advocate for tackling poverty.
- Clinics must consider how they can
  - reduce the impact of financial background on access to services and patient outcomes.
  - increase uptake of early intervention services.
  - reduce the financial burden of healthcare on low-income households.
  - demonstrate a commitment to inclusive, accessible services. equity of access and equity of management/treatments.

| Access to other specialist services for complex multisystem allergic disease |
| --- |

- Children/young people often have multiple allergic diseases, which can require input from other healthcare professionals.
- Allergy clinics should therefore have close links with other specialist services, which should be documented in a service delivery plan.

| *Working within a regional network to ensure access to specialist services* |
| --- |

- Specialist centres should support a local network to ensure access of children/young people to specialist allergy services.
- Less-specialist centres (e.g. based in “District General Hospitals” should also contribute to local networks, to ensure that are aware of specialist treatments and when to refer patients for these.

| **EDUCATION** |
| --- |

| Standardised minimum training for specialist allergy (medical/nursing/ dietetic) staff |
| --- |

- Staff must have sufficient specialist training according to national standards (where available).

| Minimum requirement for MDT staff to maintain specialist status through allergy-related CPD |
| --- |

- Is this distinct to “standardised minimum training” above?

| Empowering patients/public through knowledge/training |
| --- |

- Is this included under “Family/patient-led care with shared decision-making” or should this be a separate standard?

| Providing training for non-specialist healthcare professionals (HCPs) to support integrated care model |
| --- |

- Should local services provide training for non-specialist Healthcare Professionals including those working in community services and primary care, to support integrated care?

| *Training of students/trainees in recognition and management of allergies* |
| --- |

- Should accredited services be required to support training of students and doctors-in-training?

| *Research links* |
| --- |

- Clinical services associated with active research may be more up-to-date with the latest evidence and good practice guidelines. Should links to research be a key standard, and if so, how can this be applied to those clinics without active research teams?
